# Perinatal Outcomes of Small for Gestational Age Neonates Born With an Isolated Single Umbilical Artery

**DOI:** 10.3389/fped.2019.00079

**Published:** 2019-03-19

**Authors:** Maayan Blum, Adi Y. Weintraub, Yael Baumfeld, Reut Rotem, Gali Pariente

**Affiliations:** ^1^Department of Obstetrics and Gynecology, Soroka University Medical Center, Ben-Gurion University of the Negev, Beer-Sheva, Israel; ^2^Department of Obstetrics and Gynecology, Shaare Zedek Medical Center, Affiliated with the Hebrew University Medical School of Jerusalem, Jerusalem, Israel

**Keywords:** small for gestational age, isolated umbilical artery, perinatal mortality, preterm delivery, outcomes

## Abstract

**Objective:** To investigate pregnancy outcomes of small for gestational age (SGA) neonates born with isolated single umbilical artery (iSUA) compared to SGA neonates without iSUA.

**Study Design:** This was a population-based retrospective cohort analysis. The study group was defined as a singleton SGA neonate born with iSUA, while an SGA neonate without iSUA comprised the comparison group. We evaluated adverse perinatal outcomes in all SGA neonates born at the Soroka University Medical Center between the years 1998–2013. Multiple gestations, fetuses with known congenital malformations or chromosomal abnormalities and patients with lack of prenatal care were excluded from the study. Multivariate logistic regression models were constructed to identify independent factors associated with adverse perinatal outcomes.

**Results:** Of 12,915 SGA deliveries, 1.2% (162) were complicated with iSUA. Women in the study group were older with a significantly lower gestational age at delivery compared with the comparison group. Rates of women who conceived after infertility treatments were higher in the study group. Additionally, patients in the study group had significantly higher rates of preterm deliveries, placental abruption, cord prolapse, non-reassuring fetal heart rates and cesarean delivery were noted in the study group. These neonates had a significantly lower birth weight (1988.0 ± 697 vs. 2388.3 ± 481 *p* < 0.001) and higher rates of low APGAR scores at the first and fifth minutes after birth compared with controls. Perinatal mortality was also found to be significantly higher among SGA neonates complicated with iSUA. Preterm delivery as well as perinatal mortality were found independently associated with iSUA among SGA neonates (aOR 4.01, 95% CI 2.88–5.59, aOR 2.24, 95% CI 1.25–4.01, respectively).

**Conclusion:** SGA pregnancies complicated with iSUA are at higher risk for adverse pregnancy and perinatal outcomes as compared to SGA pregnancies without iSUA.

## Introduction

The umbilical cord is formed between the 13 and 38th day following conception and contains two umbilical arteries and one umbilical vein (1). Single umbilical artery (SUA) is defined as the absence of one umbilical artery and is considered the most common macroscopic anomaly of the placenta and the most common malformation of the umbilical cord (2). SUA can occur due to aplasia or as a consequence of atrophy of one of the arteries (3). The incidence of SUA is 0.2–1.6% in euploid fetuses compared with 9–11% in aneuploid fetuses. The reported prevalence of SUA is 4.6% of twin births and 1% of singletons (3, 4). SUA is defined isolated SUA (iSUA), if no additional chromosomal or structural abnormalities occur (5). Fetuses with an iSUA are at increased risk for intra-uterine growth restriction (IUGR) during pregnancy and small for gestational age (SGA) at birth (4, 6).

SUA has been associated with various malformations and pathologies such as cardiac malformations, cleft lip or palate, esophageal atresia, ano-rectal atresia/stenosis (7) and adverse pregnancy outcomes such as preterm birth, diabetes, epilepsy, preeclampsia, polyhydramnios, and oligohydramnios (3). The incidence of chromosomal defects in fetuses with SUA is considerably higher, specifically noting trisomy 18 with a 7-fold increased risk (8). SUA is rare in Mendelian disorders but relatively frequent in idiopathic anomalies (9). iSUA has been reported as an independent risk factor for perinatal mortality(10), cesarean section (11) and adverse pregnancy outcomes (12). Hence induction of labor at 40 weeks of gestation for iSUA fetuses has been recommended (10).

By definition, 10% of all live births are born SGA. Neonates are considered small for gestational age if they are smaller than the 10th percentile with regard to the anthropometric index being used (13). SGA neonates in comparison with appropriate for gestational age (AGA) neonates are considered to have an increased risk of complications such as bronchopulmonary dysplasia and adverse neurodevelopmental outcomes, as well as adverse pregnancy outcomes (14). Gutvirtz et al. presented iSUA as an independent risk factor for adverse perinatal outcomes in term neonates with normal estimated fetal weight prior to delivery (12). In our study, we focused on SGA neonates born with iSUA since little is known regarding this specific combination. The goal of this study was to examine the pregnancy outcomes of those SGA neonates born with iSUA compared to SGA neonates without iSUA.

## Materials and Methods

In this population-based retrospective cohort analysis, all deliveries of singleton SGA neonates occurring between the years 1998 and 2014 were included. The study was conducted at the Soroka University Medical Center (SUMC), which is the only tertiary medical center in southern Israel where virtually all births to women in southern Israel take place. Due to the diversity of this population, we believe that this cohort represents a non-selective population-based data. The institutional review board of SUMC approved the study in accordance with the Helsinki declaration (# SOR-0372-17). In complying with the Israeli Ministry of Health regulations, the institutional ethics committee did not require written informed consent because the data was obtained anonymously from medical records, with no direct participation or involvement of patients and public.

Case records of SGA neonates with iSUA were compared with SGA neonates without iSUA. The diagnosis of iSUA was confirmed following labor, as per protocol at SUMC where the midwives routinely examine the placenta and umbilical cord immediately after delivery. Thus, all cases of iSUA are confirmed by a physical examination.

In order to fit the definition of isolated SUA, we excluded from the cohort neonates that were AGA or large for gestational age neonates (LGA), multiple gestations, fetuses with known congenital malformations or chromosomal abnormalities and patients with lack of prenatal care. Those outcomes assessed included maternal characteristics, pregnancy characteristics, labor and delivery characteristics, and neonatal outcomes.

Postpartum hemorrhage is defined as the loss of 500 ml of blood following a vaginal birth, 1 L of blood following a cesarean section or a drop in the maternal hemoglobin of 3 gr% after delivery. Perinatal mortality includes one of the following occurrences: Intrauterine fetal death which is defined as the death of a fetus older than 22 weeks gestation, intrapartum death which is defined as the death of a neonate during delivery or postpartum death which is the death of a neonate 1 month after birth.

Data was collected from the computerized perinatal database of the Obstetrics and Gynecology department of the SUMC.

### Statistical Analysis

Statistical analysis was performed using SPSS 21.0 (SPSS, Chicago, IL). Initial analysis included descriptive statistics followed by advanced analytical statistics tests. Normal distributed continuous variables are presented as mean ± standard deviation, comparison between the study groups was done using *t*-test. Continuous variables which are not normally distributed are presented as median with inter-quartile range, Mann Whitney test was used for their statistical analysis. Categorical variables are presented in counts and percentages, Chi-Square or Fisher Exact test when appropriate were used. All analysis with two-sided and *p*-value of 0.05 was considered significant. Multivariate logistic regression models were constructed in order to identify independent risk factors associated with the selected outcomes.

## Results

Of 12,915 SGA deliveries that were included in the analysis, 1.2% (162) were complicated with iSUA. Table 1 presents demographic and clinical characteristics of patients delivering SGA neonates with iSUA (study group) in comparison with patients delivering SGA neonates without iSUA (comparison group). Women in the study group were older with a significantly lower gestational age at delivery. Rates of women who conceived after infertility treatments and rates of women with habitual abortions were higher in the study group.

**Table 1:** Demographic and clinical characteristics of patients delivering an SGA infant with and without iSUA.

**Characteristics**	**SGA with iSUA (*n* = 162)**	**SGA without iSUA (*n* = 12,915)**	***P*-values**
Maternal age years (mean + SD)	28.14 ± 5.82	27.43 ± 5.95	0.04
Gravidity *n* (%)1 2–4 5≤	52 (32.1) 65 (40.1) 45 (27.8)	4,067 (31.5) 5,562 (43.1) 3,282 (25.4)	0.71
Parity *n* (%)			0.37
1	64 (39.8)	4,765 (36.9)	
2–4	62 (38.5)	5,675 (44.0)	
5≤	35 (21.7)	2,472 (19.1)	
Gestational age at delivery (mean + SD)	36.80 ± 3.80	38.60 ± 2.65	<0.001
Previous cesarean delivery *n* (%)	22 (13.6)	1,614 (12.5)	0.68
BOH *n* (%)	8 (4.9)	608(4.7)	0.85
Habitual abortion *n* (%)	15 (9.3)	684 (5.3)	0.03
Pregnancy after infertility treatment *n* (%)	15 (9.3)	334 (2.6)	<0.001
Gender *n* (%)Male Female	64 (39.5) 98 (60.5)	4,723 (36.6) 8,192 (63.4)	0.44

*SGA, small for gestational age; iSUA, isolated umbilical artery; BOH, bad obstetric history*.

Table 2 compares gestational characteristics and pregnancy complications of pregnancies complicated with SGA neonates with and without iSUA. Pregnancies in the study group had significantly higher rates of preterm deliveries (37.7 vs. 12.2%, *p* < 0.001) and amniotic fluid abnormalities (10.5 vs. 2.3%, *p* < 0.001 and 13.0 vs. 8.4% *p* = 0.04 for polyhydramnios and oligohydramnios, respectively) as compared to pregnancies of the comparison group.

**Table 2:** Characteristics of pregnancy complications of SGA neonates with and without iSUA.

**Characteristics**	**SGA with iSUA *n*= 162**	**SGA without iSUA *n*= 12,915**	***P*-values**
Preterm deliveries *n* (%)	61 (37.7)	1,579 (12.2)	<0.001
Pre-gestational and gestational diabetes mellitus *n* (%)	8 (4.9)	467 (3.6)	0.37
Amnion fluid abnormalities Polyhydramnios *n* (%) Oligohydramnios *n* (%)	17 (10.5) 21 (13.0)	302 (2.3) 1,087 (8.4)	<0.001 0.04
Placenta Previa *n* (%)	2 (1.2)	60 (0.5)	0.16
PROM *n* (%)	8 (4.9)	1,048 (8.1)	0.14
Hypertensive disorders in pregnancy *n* (%)	23 (14.2)	1,347 (10.4)	0.12

*SGA, small for gestational age; iSUA, isolated umbilical artery; PROM, premature rupture of membranes. Hypertensive disorders in pregnancy included chronic hypertension, preeclampsia and severe preeclampsia*.

Labor and delivery characteristics and neonatal outcomes of the two groups are presented in Table 3. Pregnancies in the study group had higher rates of placental abruption (4.3 vs. 1.5%, *p* = 0.01), cord prolapse (4.3 vs. 0.5%, *p* < 0.001), non-reassuring fetal heart rate patterns (8.6 vs. 4.8% *p* = 0.02) and cesarean section (36.4 vs. 20.3% *p* < 0.001). These neonates had significantly lower birth weight (1988.0 ± 697 vs. 2388.3 ± 481 *p* < 0.001) and higher rates of low APGAR score at the first and fifth minutes after birth compared with the comparison group. Perinatal mortality was also significantly higher in SGA neonates complicated with iSUA (12.3 vs. 3.3%, *p* < 0.001). Induction of labor was found to be higher in the study group (16%) compared to the comparison group (11.4%), though this was not found to be significant (*p* = 0.06).

**Table 3:** Labor and delivery characteristics and neonatal outcomes of patients delivering an SGA infant with and without iSUA.

**Characteristics**	**SGA with iSUA *n*= 162**	**SGA without iSUA *n* = 12,915**	***P*-values**
Placental abruption *n* (%)	7 (4.3)	192 (1.5)	0.01
Cord prolapse *n* (%)	7 (4.3)	61 (0.5)	<0.001
Meconium in amniotic fluid *n* (%)	27 (16.5)	2,383 (18.5)	0.56
Induction of labor *n* (%)	26 (16)	1,469 (11.4)	0.06
Mode of deliver Cesarean section *n* (%) Vacuum *n* (%)	59 (36.4) 8 (4.9)	2,618 (20.3) 452 (3.5)	<0.001 0.32
Non-reassuring fetal heart rate *n* (%)	14 (8.6)	615 (4.8)	0.02
PPH *n* (%)	1 (0.6)	59 (0.5)	0.76
Infant characteristics Birth weight gr (Mean ± SD) APGAR 1 min < 7 *n* (%) APGAR 5 min < 7 *n* (%) PH < 7 *n* (%)	1988.00 ± 697.88 32 (19.6) 10 (6.2) 0 (0.0)	2388.33 ± 481.22 1,389 (10.8) 297 (2.3) 6 (0.05)	<0.001 <0.001 <0.001 1.00
Perinatal mortality *n* (%)	20 (12.3)	420 (3.3)	<0.001
Gender (%)Male Female	64 (39.5) 98 (60.5)	4,723 (36.6) 8,192 (63.4)	0.44

*SGA, small for gestational age; iSUA, isolated umbilical artery; SD, standard deviation; PPH, postpartum hemorrhage*.

In order to assess whether the increased risk for preterm delivery was independently associated with iSUA, a multivariate logistic regression model controlling for maternal age, fertility treatments and placental abruption was constructed, with preterm delivery as the outcome variable (Table 4). iSUA, was found to be independently associated with preterm delivery (adjusted OR 4.01, 95% CI 2.88–5.59, *p* < 0.001, Table 4).

**Table 4:** Multivariate logistic regression for the prediction of preterm delivery.

**Variable**	**OR**	**95% CI**	***P*-value**
iSUA in SGA neonate vs. no iSUA in SGA neonate	4.01	2.88–5.59	<0.001
Maternal age	1.03	1.02–1.03	<0.001
Fertility treatment	1.92	1.48–2.50	<0.001
Placental abruption	8.51	6.36–11.39	<0.001

*OR, odds ratio; CI, confidence interval; SUA, single umbilical artery; SGA, small for gestational age*.

Another multivariate logistic regression model was constructed, controlling for gestational age at birth, to estimate an independent association with perinatal mortality (Table 5). iSUA was found to be independently associated with perinatal mortality (adjusted OR 2.24, 95% CI 1.25–4.01, *p* = 0.01, Table 5).

**Table 5:** Multivariate logistic regression for the prediction of perinatal mortality.

**Variable**	**OR**	**95% CI**	***P*-value**
iSUA in SGA neonate vs. no iSUA in SGA neonate	2.24	1.25–4.01	0.01
Gestational age at delivery	0.69	0.67–0.70	<0.001

*OR, odds ratio; CI, confidence interval; SUA, single umbilical artery*.

## Discussion

In this large population based cohort study, there were 162 neonates born with iSUA, which is 0.01% of the entire population of the study. This rate is lower than the incidence of SUA reported in the literature [0.02–1.6% (4)], possibly due to the latter referring to the rate of non-isolated SUA. Delivery of an SGA neonate born with iSUA was found to be an independent risk factor for preterm delivery and perinatal mortality. As previously mentioned, our discoveries show a substantially higher rate of preterm delivery and perinatal mortality in SGA neonates complicated with iSUA.

Friebe-Hoffmann et al. demonstrated that 5.1% of neonates born with an isolated single umbilical artery had been delivered preterm (15). Others have reported a preterm rate of 11.8% (16). Both rates are significantly lower than in this study (37.7%) who were born preterm and had both iSUA and SGA. Battarbee et al. showed an increased incidence of preterm birth among pregnancies with iSUA emphasizing the significant increase of preterm deliveries due to medical indications, which may be explained by their findings of increased incidence of gestational hypertension and preeclampsia amongst these pregnancies (17). This, along with the tendency of obstetricians to induce labor earlier in pregnancies with fetuses who are suspected to be small for gestational age [as mentioned by Naveiro-Fuentes et al. (16)], can explain the higher rates of preterm deliveries in the present study. In this study SGA neonates with iSUA had a higher rate of induction of labor compared to SGA neonates without iSUA, thought this was not found to be significant. Tul et al. suggest that SGA fetuses are delivered earlier due to maternal conditions such as the increased incidence of hypertensive disorders (18), which may cause a hostile environment for the fetus.

We found that 36.4% of SGA neonates with iSUA had been delivered via cesarean section compared with 20.3% of SGA neonates without iSUA. Raio et al. demonstrated a reduction of Wharton's jelly in cases of SUA (19). This reduction may cause the umbilical cord to be more vulnerable to compression, leading it to be more susceptible to interruptions in blood flow during contractions compromising fetal oxygenation, thus possibly explaining the higher rate of cesarean deliveries as well as the higher rates of perinatal mortality.

Furthermore, Gutvirtz et al. demonstrated a perinatal mortality rate of 2.5% among neonates born with iSUA (12), lower than was demonstrated in our study group. Another possible explanation for the higher rates of perinatal mortality in our study, relates to structural deviations of the cord which may elevate the risk of umbilical cord accidents (12). Placental pathology can be a cause for both fetal growth restriction and perinatal mortality (20, 21), thus may account at least in part for the increased rate of perinatal mortality. Francis et al explain a higher rate of perinatal mortality in term and post term SGA neonates due to placental “aging” (22).

While an increased risk of perinatal mortality in SGA neonates has been demonstrated in many studies (20, 22–24), this study is the first to look at the combination of both SGA and iSUA. These results ascertain the importance of studying these comorbidities proving these neonates to have an increased risk of developing adverse outcomes compared with neonates complicated with iSUA alone or SGA alone.

Battarbee et al. hypothesized that the cause for the higher rate of neonates with SUA being born SGA is due to the fact that they are at greater risk of abnormal placental development and perfusion (17). Bugatto et al. suggest that it is a consequence of disorders in maternal-placental circulation rather than placental insufficiency (25).

Recent studies in the field of epigenetics discuss the association between maternal environmental exposures and offspring outcomes, specifically offspring growth. Nagarajan et al. researched the effect of maternal stress on neonatal birth weight (26). They demonstrated that HSD11b2 promoter methylation, a key component in the cortisol binding pathway, was significantly higher in neonates clinically diagnosed with intrauterine growth restriction (IUGR). Kitsiou-Tzeli et al. speak of maternal nutrition, exposure to toxic substances and changes in the *in-utero* expression of maternally imprinted genes and their effect on neonatal and infant growth (27). One study reported on mutations in oncogenes such as PLAG1, a key factor in the IGF2 pathway, resulting in IUGR fetuses (28). Due to this, we hypothesize that such epigenetic mutations can also be expressed as an SUA. Further research must be done in this field focusing on SGA and SUA since this may contribute to our understanding of the perinatal outcomes discussed above.

The main strength of our population-based study is the large sample size allowing us to draw conclusions regarding delivering SGA neonates with and without iSUA. Additionally, this large sample size allowed us to study a specific smaller population (namely SGA neonates complicated by iSUA) in our population and its association with several clinically significant outcomes. Nevertheless, our study has a number of limitations mainly due to its retrospective design, including the potential of missing data. It must be noted that the data was reported by an obstetrician directly after delivery and was routinely reviewed by skilled medical secretaries prior to entering it into the database. Coding was done after assessing the medical prenatal care records together with the routine hospital documents. Hence this makes this potential source of selection bias less plausible.

In conclusion, giving birth to an SGA neonate with iSUA is associated with adverse pregnancy outcomes. It is imperative to perform Intensive fetal monitoring in the presence of iSUA during delivery of a fetus suspected to be born SGA in order to decrease perinatal morbidity and mortality. Researching this particular group of neonates can be of great significance in aiding the medical team in their decision making during pregnancy and delivery of these fetuses and may possibly help prevent severe deleterious outcomes.

## Data Availability

The datasets for this manuscript are not publicly available because hospital policy. Requests to access the datasets should be directed to adiyehud@bgu.ac.il.

## Author Contributions

MB, AW, YB, RR, and GP: conception, planning, carrying out, analyzing, and writing up the work.

### Conflict of Interest Statement

The authors declare that the research was conducted in the absence of any commercial or financial relationships that could be construed as a potential conflict of interest.
